# Low-grade oncocytic tumor of kidney: A case report

**DOI:** 10.1097/MD.0000000000046256

**Published:** 2025-12-26

**Authors:** Ting-Shuo Kang, Yi-Zhong Chen

**Affiliations:** a Division of Urology, Department of Surgery, Changhua Christian Hospital, Changhua, Taiwan.

**Keywords:** oncocytic, oncocytoma, renal tumor

## Abstract

**Rationale:**

Low-grade oncocytic tumor (LOT) is characterized by cells with oncocytic/eosinophilic cytoplasm. Despite the clear definition of the tumor, challenges persist as some tumors resemble LOT, complicating the diagnosis. Therefore, understanding its characteristics is crucial for accurate diagnosis and differentiation from other renal oncocytic tumors.

**Patient concerns:**

An 81-year-old woman was incidentally found to have a right renal mass during routine examination.

**Diagnoses:**

Histological examination of the specimen obtained from a partial nephrectomy revealed oncocytic cells with abundant eosinophilic granular cytoplasm, indistinct cell borders, uniform round nuclei, and inconspicuous nucleoli, arranged in small nesting and trabecular patterns. A vague peri-nuclear halo was also observed. Immunohistochemistry demonstrated significant positivity for CK7, while CD117, CA9, GATA3, CK20, RCC, and GPNMB were negative. Based on these findings, we diagnosed the tumor as a LOT.

**Interventions:**

The patient underwent partial nephrectomy for tumor removal.

**Outcomes:**

There was no evidence of recurrence or metastasis during follow-up.

**Lessons:**

LOT can exhibit variations in its immunohistochemical profile. Therefore, additional studies and the accumulation of similar cases are necessary.

## 1. Introduction

Diagnosing oncocytic/eosinophilic renal tumors can be challenging due to their heterogeneity. Even with advanced immunostaining techniques, definitive diagnosis of well-defined entities can remain elusive. Moreover, several new entities in this category have been described recently.

Low-grade oncocytic tumor (LOT) was introduced as a new entity, and several studies have reported its immunohistochemical features, showing positive CK7 and negative CD117 results. Despite clear diagnostic criteria, some tumors mimic LOT, complicating diagnosis.

## 2. Case presentation

A right renal tumor was detected incidentally on abdominal computed tomography (CT) over a 3-year period in an 81-year-old woman. She was asymptomatic and initially opted for observation in 2020, presenting to our hospital in 2023. The abdominal CT revealed a right renal tumor.

**Figure 1.**
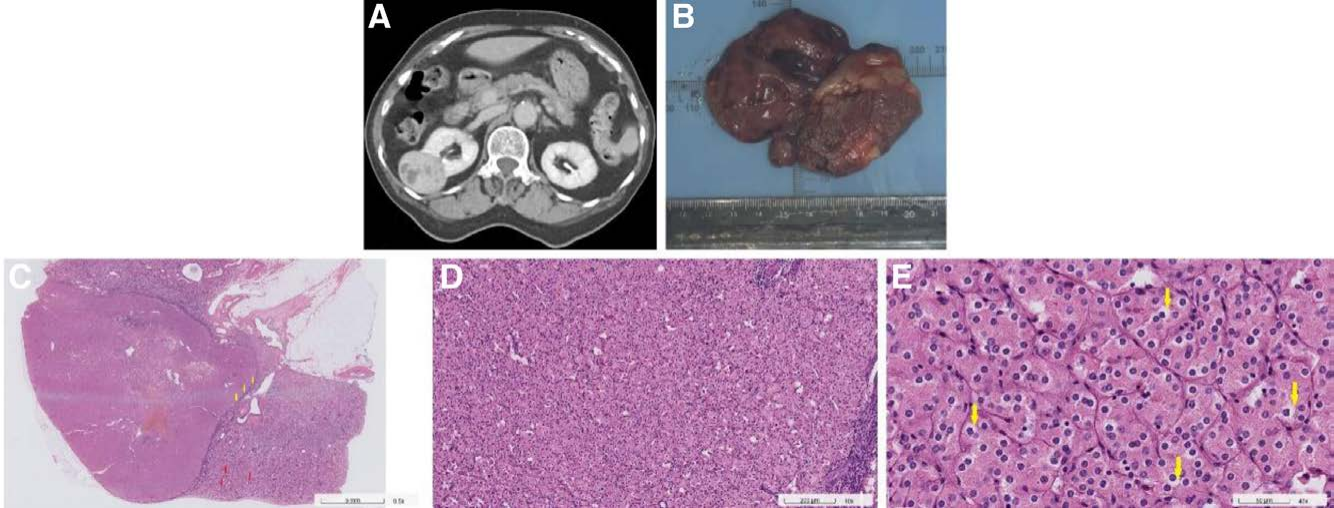
Radiological and histological findings. Contrast-enhanced CT demonstrated early tumor enhancement (A). Grossly, the cut surface appeared solid (B). Histology showed a sharply demarcated tumor (yellow arrows) with cortical involvement (red arrows) (C; H&E, 5×), composed of oncocytic cells (D; H&E, 100×) with uniform round nuclei and peri-nuclear halos (yellow arrows) (E; H&E, 400×). CT = computed tomography.

Abdominal CT showed a 5-cm right interpolar renal tumor (previously 4 cm 3 years prior) with early contrast enhancement (Fig. 1A). Due to tumor progression, partial nephrectomy was performed.

**Figure 2.**
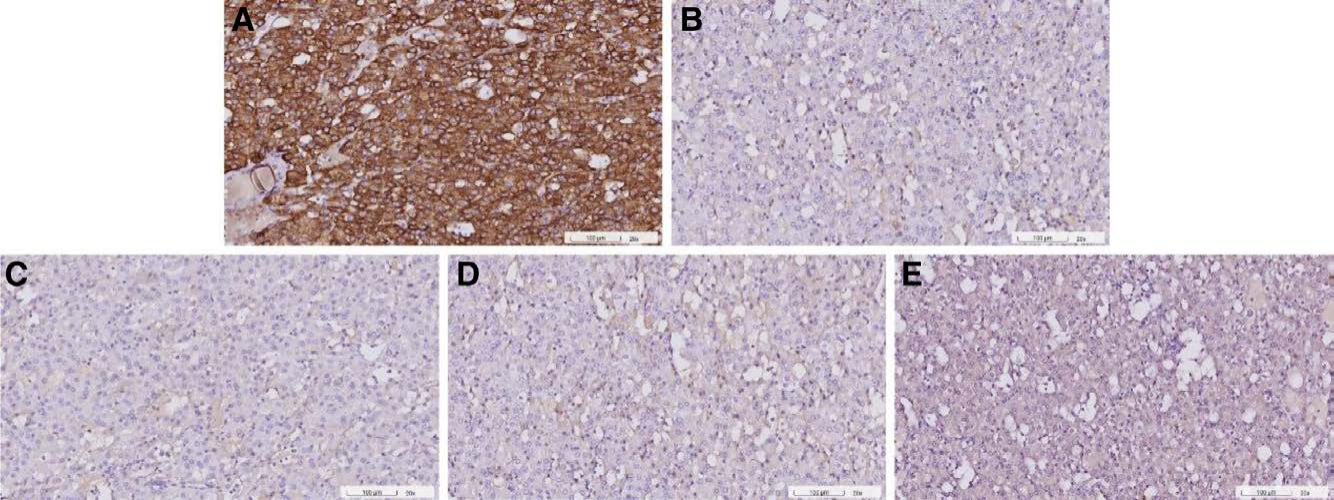
Immunohistochemical staining profile. The oncocytic tumor cells were diffusely positive for CK7 (A), while negative for CD117 (B) and CK20 (C). This immunohistochemical profile was incompatible with a chromophobe renal cell carcinoma or an oncocytoma, which characteristically demonstrates CD117 positivity. A diagnosis of eosinophilic solid and cystic renal cell carcinoma, which usually demonstrates patchy immunoreactivity for CK20 and at most focal CK7 positivity, was also unlikely. CAIX (D) and RCC (E) were negative, therefore excluding a low-grade clear cell renal cell carcinoma. Appropriate positive and negative controls were run for all antibodies tested on this tumor (not shown).

The resected tumor weighed 46.8 g and measured 5.8 × 4 × 2 cm in size. The tumor was well-demarcated, tan-yellow, and elastic, bulging the renal surface (Fig. 1B). On sectioning, it involved the renal cortex and was 0.3 cm from the parenchymal margin (Fig. 1C). Microscopically, the tumor comprised oncocytic cells with abundant eosinophilic granular cytoplasm, indistinct cell borders, uniform round nuclei, and inconspicuous nucleoli, arranged in small nests and trabeculae (Fig. 1D). Vague peri-nuclear halos were also noted (Fig. 1E). Immunohistochemistry showed diffuse strong CK7 positivity (Fig. 2A); CD117 (Fig. 2B), CK20 (Fig. 2C), CA9 (Fig. 2D), GATA3, and RCC (Fig. 2E) were negative. GPNMB was strongly positive, suggesting mTOR pathway activation. FH and SDHB showed intact cytoplasmic expression. Based on these findings, the tumor was diagnosed as LOT. The patient was discharged without complications on postoperative day 6. A 6-month follow-up CT showed no recurrence or metastasis.

## 3. Discussion

Recent WHO classifications (2022) include new oncocytic/chromophobe-like entities such as LOT and eosinophilic vacuolated tumor.^[1]^ Differential diagnoses include eosinophilic chromophobe RCC, oncocytoma, SDH-deficient RCC, eosinophilic solid and cystic RCC, and fumarate hydratase-deficient RCC.

Gholami et al reported that among contrast-enhancing renal masses ≤ 4 cm, approximately 20% were benign (e.g., oncocytoma, angiomyolipoma).^[2]^

LOTs are typically solitary, solid, and nonsyndromic. Histologically, they show solid and nested growth with uniform round to oval nuclei and focal peri-nuclear halos. Immunohistochemically, they are consistently CK7+/CD117- and negative for CA9 and CK20, but positive for FH.^[3–5]^ Williamson et al additionally reported GATA3 positivity and TSC/MTOR pathway alterations in 17 LOTs.^[4]^ Akgul et al confirmed these findings in 23 cases, with SDHB positivity in all tested cases.^[5]^

Our case shared typical LOT features: solitary, solid, tan-yellow tumor with uniform nuclei and peri-nuclear halos in nested/trabecular patterns. The immunohistochemical profile (CK7+/CD117-) was consistent. However, unlike most reported LOTs,^[4,6,7]^ our case was GATA3-negative. Strong GPNMB positivity indicated mTOR pathway activation, which is characteristic of LOT^[8]^ and associated with MTOR mutations.^[9,10]^ We diagnosed the tumor based on the current understanding of LOT.

LOTs are usually sporadic and indolent, without age or gender predilection.^[3,5,6]^ To date, no progression or metastasis has been reported.^[3,6]^ However, this study is limited by its single case design and short follow-up period, which may restrict the generalizability of the findings. Due to limited data, larger cohorts with long-term follow-up are needed to establish surveillance protocols.^[5]^

Some oncocytic/eosinophilic renal tumors remain unclassifiable. Ongoing genetic research may refine classifications. Despite diagnostic challenges, detailed histological reporting is essential for understanding these evolving entities.

## Author contributions

**Investigation:** Ting-Shuo Kang.

**Writing – original draft:** Ting-Shuo Kang.

**Writing – review & editing:** Yi-Zhong Chen.
